# Reviewers acknowledgement

**DOI:** 10.1002/2211-5463.13505

**Published:** 2022-12-01

The editors of *FEBS Open Bio* would like to thank all those who have given their time and expertise to review articles submitted for publication in Volume 12. Although *FEBS Open Bio* does not seek to judge the importance of submissions, our reviewers carefully scrutinize the experimental design and results of all papers and challenge authors about their conclusions.

The names of these reviewers are listed below; we apologize if we have inadvertently omitted anyone.

Meghdad Abdollahpour‐Alitappeh

Rashidah Abdul Rahim

Wolf‐Rainer Abraham

Rosa Aghdam

Mark Agostino

Sebastian Aguayo

Rodrigo Aguiar

Hosseini Ahmad Zavaran

Ayodele Alaiya

Réka Albert

Elisabetta Aldieri

Johari Mohd Ali

Malcolm Alison

David S. Allan

Peter Alston

Monica Amblar

Emmanuel Ampofo

Paolo Arosio

Avraham Ashkenazi

Tudor Constantin Badea

Gwenael Badis‐Breard

Marica Bakovic

Kiran Bali

Matthew Banton

Sebastien Banzet

Helene Barelli

Gonzalo Barriga

Rafał Bartoszewski

Alexei Basnakian

Zsófia Bata

Alzir Batista

Christine Belloir

Alexandre Benedetto

Laureline Berthelot

Francesco Berti

Alfredo Berzal‐Herranz

Dharmendra K. Bhargava

Arindam Bhattacharjee

Jana Biermann

Saikat Biswas

Ulrich Axel Bommer

Gloria Borgstahl

Luis Miguel Botana López

Evzen Boura

Salvatore Bozzaro

Philip N. Bryan

Viljemka Bučević Popović

Pamela Burger

Boudewijn M.T. Burgering

Hamilton Cabral

Erica P. Cai

Lukas Cajanek

Philip C. Calder

Vincenzo Calvanese

Laura Calvo

Ihosvany Camps

Michael J. Caplan

Anthony Carlos

Pietro Carotenuto

John A. Carver

Filipe Castro

Silver Ceballos

Patrick Celie

Carolina Centeno

Kiran Chada

Kausik Chakrabarti

Joydeep Chakraborty

Sohini Chakraborty

Walee Chamulitrat

Mark Chappell

Lucienne Chatenoud

Max Chavarria

Cédric Chaveroux

Jie Chen

Yeh Chen

Quan Cheng

John Chilton

Elisabeth S. Christians

Steven L. Cobb

Kelly Coffey

Martine Collart

Laura Corrales

Paola Costelli

Christine Cremo

Peter Cresswell

Jean Luc Darlix

Ramanuj DasGupta

Mads Daugaard

Alberto Davalos

Marina Degoricija

Esmerilda Delicado

Marion Delphin

Elena Demidova

Selami Demirci

Evgeny V. Denisov

Jiahui Ding

Yu Ding

Manjusha Dixit

Ellie Dommett

Stanislav Drapela

Yang Duan

Gabriel Duette

M. Beatriz Durán‐Alonso

Dibyendu Dutta

Sabine Ehrt

Elis Eleutherio

Anna Ermund

Genevieve Escher

Joao Esteves

Eliseo Eugenin

Minrui Fan

Zeba Farooqui

Suzanne Faure‐Dupuy

Vadim Fedorov

Yansheng Feng

Yingang Feng

Eva Fenyvesi

Suzanne Fergus

Stuart J. Ferguson

Rafael Fernández‐Chacón

João M. Fernandes Neto

Jarlei Fiamoncini

Arnaud Firon

Giulio Francia

Jing Fu

Naonobu Fujita

Yoshitaka Fukada

Atsunori Fukuhara

Tomoyuki Furuyashiki

Matthias Futschik

Márcia Garcez

Carmen C. Garcia

Brigitte Gasser

Maria Gazouli

Róna Gergely

Marta Giacomello

Ying Gong

Carlos Gonzalez

Daniel R. Gonzalez

Miguel Angel Gonzalez

Pablo Gonzalez

Marina Govoroun

Darrell Green

Elvira V. Grigorieva

Christian Gruber

Henri Gruffat

Paolo Grumati

Wenchao Gu

Alejandra Guerra‐Castellano

Dongsheng Guo

Kararzyna Gurzawska‐Comis

Toshio Hakoshima

Gentzon Hall

Marshall Hampton

Anis Hanna

Takeshi Harayama

Takeshi Hase

Kazuhiko Hashimoto

Lukas J. A. C. Hawinkels

Yoshiki Hayashi

László Henn

Simon Hennig

Andrea Herrero‐Cervera

Hidekazu Hiroaki

Kohji Hizume

Jörg Höhfeld

Marcel Hollenstein

Suguru Honda

Tian Hong

Vivian Y.H. Hook

Tamarisk Kerry Horne

Gabriel Hornink

Guy Howard

Chao‐Kai Hsu

Yingbo Huang

Veronica Huber

Tanweer Hussain

Mahmoud A. A. Ibrahim

Didem Ilter

Takeshi Imai

Yuuki Imai

Ikbal Agah Ince

Irina Ingold

Ivan Iourov

Esma R. Isenovic

Kosei Ito

Tohru Itoh

Ivan Ivanov

Makoto Iwashima

Venkatakrishna R. Jala

Eric Jan

Elisa Helena Farias Jandrey

Taeck JoongJeon

Esque Jeremy

Peihua Jiang

Rilei Jiang

Xin Jin

Mohit Kumar Jolly

Stefanie Jonas

Malene Jørgensen

Walter Kahr

Mohammad Kamran

Dmitry Sergeevich Karpov

Hiroyuki Katoh

Benjamin Kaupp

Özgecan Kayalar

Zeliha Keskin Alkaç

Gholamreza Khaksar

Bilon Khambu

Sang Geon Kim

Wanil Kim

Yun Hak Kim

Chandra Kishore

Kaio Kitazato

Prasat Kittakoop

Nancy Klauber‐DeMore

Ingeborg Klymiuk

Andrzej Kochanski

Minna Konert

Matthijs Kox

Peter Kristensen

Donald Kuhn

Tracey Kuit

Hitoshi Kurose

Qi Lai

Sowmya Lakshmi

Ingrid Lang

Göran Larson

H. Peter Larsson

Douglas Laurents

Ahmed Lawan

Sunil Laxman

Karine G. Le Roch

Djamel Lebeche

Hangmao Lee

Hakon Leffler

Roger Leng

Olivia Lenoir

Jiao Li

Mingli Li

Wei Li

Yen‐Liang Li

Yifei Li

Yue‐Feng Li

Zhenfei Li

Zha Liangping

Sara Liin

Keesiang Lim

Sai Kiang Lim

Fabio Mitsuo Lima

Ganbg Liu

Tong Liu

Beata Lontay

Marcelo Lopez‐Lastra

Guillermo López‐Lluch

Svenja Lorenz

Ricardo O. Louro

Kefeng Lu

Qingchun Lu

Giuseppe Lucarelli

Anastasios Lymperopoulos

Cong Ma

Harold MacGillavry

Peter Macheroux

Mateusz Maciejczyk

Anne‐Marie Madec

Andrea Magrì

Kasturi Mahadik

Bibekanand Mallick

Anitha Mamillapalli

Benedicte Manoury

Edward Manser

Luis Manuel Peña‐Rodríguez

Arul Marie

Mija Marinković

Mozart Marins

Alfredo Martinez

Jonathan Martinez‐Fabregas

Claudia Martini

Silvia Martini

Sabata Martino

Amanda Martins Baviera

Rute Gonçalves Matos

Toshiro Matsui

Yoshiyuki Matsuo

Maria Dolores Mayan Santos

Owen J.T. McCarty

Peter N. Meissner

Thomas Melendy

Alessandro Mereu

Akira Mima

Kitti Mintál

Simone Mirabilii

Shohei Mitani

Debashis Mitra

Zsófia Molnár

Renato Monteiro

Michael N. Moore

Masahiro Morita

Yoshinori Moriyama

Dina Mostafa

Lyndsey Muehling

Christian Muench

Raphael Munoz

Gabriela Murillo

Lubna Nadeem

Koji Nagata

Istvan Nagy

Zsolt István Németh

Hans Neubauer

Thong Ba Nguyen

Michael Niepmann

Kacper Nijakowski

Keisuke Nimura

Wenyan Niu

Kinga Nyiri

Chiaki Ogino

Chika Ogura

Miho Ohsugi

Koichi Ojima

Yasushi Okamura

Valentyn Oksenych

Heidi Olzscha

Jasminka Omerovic

Joseph Thomas Opferman

Edgardo Ortiz

Tadayuki Oshima

Lado Otrin

Kazuhisa Ozeki

Petar Ozretić

Nigel Page

Ashutosh Pandey

Mark S. Parcells

Norbert Pardi

Arli Aditya Parikesit

Krzysztof Pastuszak

Paola Patrignani

Luigi Michele Pavone

Helen Pearson

Luiz Penalva

Mario Pende

Jacques Le Pendu

Juan J. Perez

Gonzalo Pérez‐Mejías

Milica Perišić Nanut

Thomas Peters

A. Petry

Astrid Pinzano

Zoi Piperigkou

Marie Pohl

Indira D. Pokkunuri

Guillaume Postic

Anna Przybylska‐Piech

Rafael Pulido

Daniela Quaglino

Jon Rainford

Devesh Raizada

Joao Ramalho‐Santos

Qitao Ran

Basabi Rana

Pablo Ranea‐Robles

Shivangi Rastogi

Eduardo Reis

Biserka Relic

Ronak Reshamwala

Andrew Resnick

Félix A. Rey

Jonathan M. Rhodes

Eduardo Rial

Jana Riegger

Kristian Riesbeck

Josep Rizo

Christian E. Rocheleau

Maurizio Romano

Sandra Romero‐Cordoba

Marianne Rooman

Jorge Luis Rosas‐Trigueros

Adhiraj Roy

Stephen Rutherford

Peter J. Sadler

Daisuke Saito

Fabio Sallustio

Mauro Salvi

Ricardo Sánchez Prieto

Motoaki Sano

Roberto Santana

Prasanna Kumar Santhekadur

Janine Hertzog Santos

Gaetano Santulli

Leandro Sastre Garzón

Ryousuke Satou

Vijay Kumar Saxena

Pauline Schaap

Michael Schrader

Juerg Schwaller

Christine Schwartz

Herbert Schwarz

Andreas Scorilas

Yusuke Sekine

Heloisa S. Selistre‐de‐Araujo

David Selva

Monika Sharma

Yaron Shav‐Tal

Jingshi Shen

Rong Shen

Varda Shoshan‐Barmatz

Bhupendra Shravage

Ana Cristina Simoes e Silva

Michel Simon

Archana Singh

Richa Singhania

Thomas Soellner

Thierry Soldati

Luis Felipe Somarribas

Nahum Sonenberg

Jiuzhou Song

Minseok Song

Yujun Song

Ann Sperry

Amulya Sreekumar

Sree Rama Chaitanya Sridhara

Robin Stanley

Robert C. Stanton

Jim Francis Staples

Elliott Stollar

Venkat Subramanian

Masakazu Sugishima

Bing Sun

Zhen Sun

Aungkura Supokawej

Laura Suter‐Dick

Hitoshi Suzuki

Michio Suzuki

Katarzyna Szkudelska

Yasuhiko Tabata

Manuel Taft

YH Taguchi

Kenya Takahashi

Markku Ilmari Tammi

Chanderdeep Tandon

Fuzhou Tang

A. I. Tarasov

Olle Terenius

Suman S. Thakur

Vengatesen Thiyagarajan

David H. Thompson

Alfred E. Thumser

Tsukasa Tominari

Signe Torekov

Takehiko Tosha

Michel L. Tremblay

David A. Tumbarello

Ben Turk

Taro Uyeda

Daniela Valensin

Priit Väljamäe

Willem J.H. van Berkel

Sander I. van Kasteren

Héctor Vázquez‐Meza

Massimo Venditti

Balázs Veres

Yash Verma

Joao B. Vicente

Bruno Victor

Ivan I. Vorobiev

Anderson Vulczak

Elmar Wahle

Eric Waltari

Cheng Wang

Jian Guang Wang

Jiangxin Wang

Leyi Wang

Lipeng Wang

Ping Wang

Michel Wassef

Helen Watson

Mark Watson

Natalie Wee

Jinhong Wei

Brian Wigdahl

Bart O. Williams

Anne Willis

Gerlof Sebastiaan Winkler

Nadine Wiper‐Bergeron

K. Witwer

Anouk Wouters

Antoni Wrobel

Yuan Seng Wu

Xue Xiao

Bing‐Cong Xing

Jianhua Xiong

Junyu Xu

Shuhei Yamada

Yasuyuki Yamada

Kensuke Yamaguchi

Atsushi Yamamoto

Atsuko Yamashita

Takahiro Yamauchi

Chen Yan

Hideyuki Yanai

Jason Yang

Wei‐Dong Yang

Masato Yano

Ninghua Yao

Tianlei Ying

Hideo Yonezawa

Seock‐Won Youn

Pengfei Yu

Ziniu Yu

Lifeng Zhang

Ruxu Zhang

Weimin Zhang

Xingxing Zang

Yanhui Zhang

Tali Zitman‐Gal

Li Zuo

Janja Zupan

